# Overexpression of Rad51C splice variants in colorectal tumors

**DOI:** 10.18632/oncotarget.3209

**Published:** 2014-12-30

**Authors:** Arjun Kalvala, Li Gao, Brittany Aguila, Tyler Reese, Gregory A. Otterson, Miguel A. Villalona-Calero, Wenrui Duan

**Affiliations:** ^1^ Comprehensive Cancer Center, The Ohio State University College of Medicine and Public Health, Columbus, Ohio, U.S.A; ^2^ Division of Medical Oncology Department of Internal Medicine, The Ohio State University College of Medicine and Public Health, Columbus, Ohio, U.S.A; ^3^ Department of Pharmacology at The Ohio State University College of Medicine and Public Health, Columbus, Ohio, U.S.A

**Keywords:** Rad51C isoform overexpression, Promoter methylation, Colorectal tumors

## Abstract

Functional alterations in Rad51C are the cause of the Fanconi anemia complementation group O (FANCO) gene disorder. We have identified novel splice variants of Rad51C mRNA in colorectal tumors and cells. The alternatively spliced transcript variants are formed either without exon-7 (variant 1), without exon 6 and 7 (variant 2) or without exon 7 and 8 (variant 3).

Real time PCR analysis of nine pair-matched colorectal tumors and non-tumors showed that variant 1 was overexpressed in tumors compared to matched non-tumors. Among 38 colorectal tumor RNA samples analyzed, 18 contained variant 1, 12 contained variant 2, 14 contained variant 3, and eight expressed full length Rad51C exclusively. Bisulfite DNA sequencing showed promoter methylation of Rad51C in tumor cells. 5-azacytidine treatment of LS-174T cells caused a 14 fold increase in variant 1, a 4.8 fold increase for variant 3 and 3.4 fold for variant 2 compared to 2.5 fold increase in WT.

Expression of Rad51C variants is associated with FANCD2 foci positive colorectal tumors and is associated with microsatellite stability in those tumors. Further investigation is needed to elucidate differential function of the Rad51C variants to evaluate potential effects in drug resistance and DNA repair.

## INTRODUCTION

The Fanconi Anemia (FA) pathway is an example of homologous recombination (HR), repairing DNA damage and maintaining genomic stability. Inherited genetic alterations of FA complementation groups result in FA, characterized by chromosomal instability, congenital musculoskeletal abnormalities, bone marrow failure and susceptibility to cancer [1]. Genetic studies have demonstrated that monoubiquitinated FANCD2 and I by an FA core complex (FANCA, B, C, E, F, G M and L) is impaired during FA [2, 3]. Once monoubiquitinated, FANCD2 and I co-localize with DNA damage response proteins such as Rad51C (FANCO) to mediate HR repair [4, 5]. Defects in Rad51C have been documented as the cause of FA complementation group O (FANCO) disorder with a similar phenotype as that seen in FA core complex disorders, including defective DNA damage repair and hypersensitivity to DNA damaging agents [6-8].

In yeast, two hybrid screening and co-immunoprecipitation studies have shown a heterologous interaction between the RAD51 paralogs forming two stable protein complexes, a dimer consisting of Rad51C-XRCC3 complex and a larger (BCDX2) complex consisting of XRCC2, RAD51B, C and D [9,10]. Rad51C is the only paralog existing in both complexes [11, 12]. *In vitro*, Rad51C is shown to have single stranded DNA-dependent ATPase activity through a pairwise interaction with RAD51B [13, 14], and also participates in resolution of holiday junction intermediates by interacting with XRCC2 and Rad51B during late stages of HR [15-17].

Alternatively spliced isoforms have been demonstrated to play different or even antagonistic biological roles compared to the full length wild type mRNA in a variety of systems [18-20]. Some of the important diseases caused by *cis* acting or *trans* acting protein splicing factors includes cystic fibrosis, dementia, premature aging and cancer [21-24]. Functional characterizations of splice variants of the DNA repair gene Rad52 have previously been shown to increase direct repeat recombination between sister chromatids exchange more often expressed in human and yeast cells [25-27]. A variant of hRad51, hRad51delta ex9, resulting from a frame shift mutation causing deletion of exon 9 has been shown to promote higher DNA strand exchange activity when compared to that of hRad51 [28]. Splice variants for Rad51d have been shown to functionally interact with BCDX2 complex in mice and mediate strand-annealing reactions during HR [29, 30].

Although screening for Rad51 founder mutations in patients with colorectal and prostate tumors have shown no mutations [31], little is known of the potential influence of splice variants of this gene in colorectal tumors. Here we report the expression of splice variants of Rad51C in colorectal tumors and cells and differences in the expression of these variants between malignant and normal phenotypes.

## RESULTS

### Rad51C splice variants in colorectal tumors

**Figure 1:**
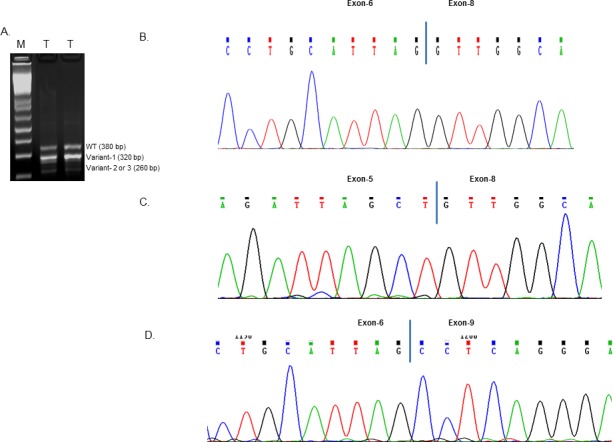
RT-PCR amplification and sequence analysis of Rad51C variants A) The Total RNA isolated from tumors (T) were RT-PCR amplified using the primers ERF and ERR as mentioned in Supplementary Table S1. The 1.5% agarose gel electrophoresis image of the amplified cDNA at 380, 320 and 260 bp fragment is shown. The amplified products were subjected to sequencing analysis that shows Rad51C. B) Variant 1 without exon-7; C) Variant 2 without exon-6 and 7 and D) Variant 3 without exon-7 and 8.

**Figure 2:**
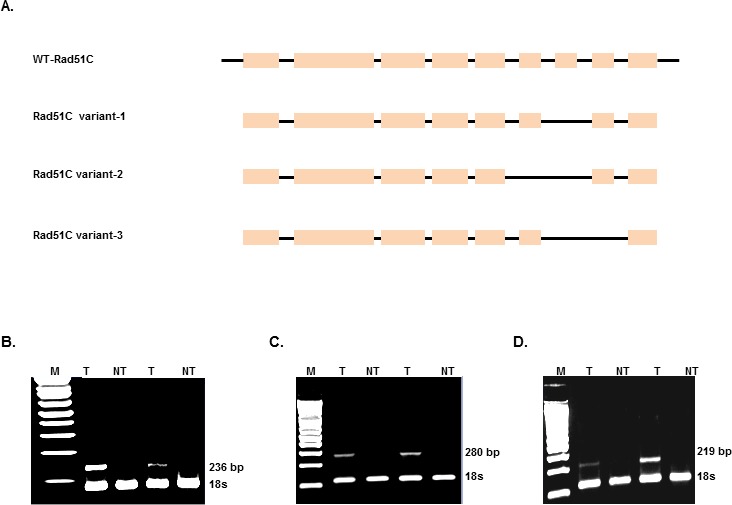
Identification of Rad51C splice variants in colorectal tumors A) Schematic representation of Rad51C variants. RNA was isolated from a total of 38 colorectal tumors (T) and corresponding non-tumor (NT) and reverse transcribed to cDNA and PCR amplified using variant specific primers (Supplementary Table S1). The electrophoresis gel image of RT-PCR amplified products showed 236 bp fragment for variant 1 (2B); 280 bp fragment for variant 2 (2C) and 219bp fragment for variant 3 (2D). 18s is loaded as internal control for each variant which is about 120 bp fragment.

To investigate the expression of Rad51C in tumors, we isolated total RNA from colorectal tumors. RT-PCR amplification of cDNA between exons 5 and exon 9 of Rad51C using the primers ERF and ERR (Supplementary Table S1) produced three products with sizes of 380 bp, 320bp and 260bp (Fig. 1A). The purified gel products were subjected to TOPO TA cloning and subjected to sequencing using universal primers. From the sequencing analysis we identified three alternatively spliced variants of the Rad51C gene. The alternatively spliced transcript variant 1 is joined from the 3′-end of exon-6 to the 5′-end of exon-8 (Fig. 1B), variant 2 is joined at the 3′- end of exon-5 by the 5′-end of exon-8 (Fig. 1C), and variant 3 is joined at the 3′-end of exon-6 with the 5′-end of exon-9 (Fig. 1D). A depiction of Rad51C short variants generated by alternative splicing is shown in Fig. 2A.

### Frequency of Rad51C variants in human colorectal tumors

To evaluate the expression of the alternatively spliced variants in colorectal tumors, we designed variant specific primers based upon the predicted sequences of each cloned variant. The forward and reverse primer sequence for variant 1, 2 and 3 with their respective amplicon sizes is shown in Supplementary Table S1. Rad51C variant-specific RT-PCR amplification is shown in Figure 2 (Fig. 2B for variant 1, Fig. 2C for variant 2, and Fig. 2D for variant 3). 18S ribosomal RNA was used as loading control.

Of the 38 colorectal tumors analyzed, 18 (47%) tumors expressed variant 1, 12 (32%) tumors had variant 2 and 14 (37%) tumors expressed variant 3. The frequency for each variant in tumors is indicated in Table 1.

**Table 1:** Expression of Rad51C variants in tumors

S.No	Variant-1	Variant-2	Variant-3
Tumors	18/38 (47%)	12/38 (32%)	14/38 (37%)
Variants expressed in FANCD2 foci negative tumors / Total negative tumors	2/12 (17%)	2/12 (17%)	1/12 (8%)
Variants expressed in FANCD2 foci positive tumors / Total tumors	16/26 (62%)	10/26 (39%)	13/26 (50%)
Fishers exact P value	0.01	0.26	0.027

### Rad51C variants are overexpressed in Tumors

**Figure 3:**
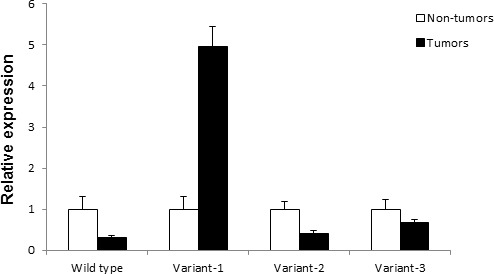
Real time PCR analysis of Rad51C variant expression in colorectal tumors and non-tumors The total RNA was isolated from 9 colorectal tumors and non-tumors and reverse transcribed to cDNA. The cDNA was then used as template for Rad51C variant expression analysis using the real time specific primers and SYBR green dye (Supplementary Table S1). On average variant 1 was expressed 4.95 fold higher as compared to matched non-tumors. Wild type, variants 2 and 3 were expressed at lower levels. The expression levels are given as relative RNA levels of each of the three variant in folds normalized by the expression level of the non-tumors.

To quantitatively analyze the differential expression of each of Rad51C variant mRNA in colorectal tumors, we compared mRNA expression levels between tumors and corresponding non-tumors using real time PCR. The real time PCR was performed using the primers as mentioned in Supplementary Table S1. These primer pairs amplify amplicons between 100bp and 200bp designed for real time PCR. The 18S ribosomal rRNA was used as endogenous control. Relative quantitative analysis of the expression of short variants showed Rad51C variant 1 is over expressed in tumors as compared to non-tumor tissue. On average, variant 1 RNA was 4.95 fold higher in tumors. Variants 2 and 3 were expressed at 2.43 fold and 1.47 fold lower in tumors comparing to non-tumor tissues (Fig. 3). In tumors, the relative expression for each variant compared with wild type Rad51C was 8.05 fold higher for variant 1, 1.81 fold higher for variant 3 and 1.72 fold lower for variant 2 (Supplementary Figure S1).

### Rad51C variants and FANCD2 foci

We next evaluated if the expression of the Rad51C variants correlates with FANCD2 foci formation. We analyzed FANCD2 foci status and Rad51C variants expression in colorectal tumors. Immunofluorescence based FATSI triple staining [32] was used to evaluate FANCD2 foci status. Among 38 tumors, 12 (32%) of samples were noted to lack FANCD2 foci and 26 (68%) tumors were FANCD2 foci positive.

Of the 12 FANCD2 foci negative tumors, Rad51C variant 1 was present in two (17%) tumors; two tumors (17%) had variant 2, and one tumor (8%) contained variant 3 (Table 2). Among the 26 foci positive tumors, Rad51C variant 1 was present in 16 (62%) tumors; 10 tumors (38%) had variant 2, and 13 tumors (50%) had variant 3 (Table 2).

**Table 2:** Analysis of Rad51C variant expression in FANCD2 Foci tumors with Microsatellite Stability and Instability

S.No	FancD2 Foci-Positive	FancD2 Foci-Negative	Total	P value
FANCD2 Foci	26	12	38	
**Microsatellite Stable (MSS)**	25	9	34	0.08
Variant-1	15	1	16	0.01
Variant-2	9	2	11	0.68
Variant-3	12	1	13	0.02
**Microsatellite Instable (MSI)**	1	3	04	0.08
Variant-1	1	1	02	0.99
Variant-2	1	0	01	0.25
Variant-3	1	0	01	0.25

MSS Indicates microsatellite stable; MSI, microsatellite instability

### Rad51C variants expression and microsatellite instability

Microsatellite instability (MSI) is a condition of genetic hypermutability that results from impaired DNA Mismatch Repair (MMR). MSI is considered an important molecular signature in colorectal cancers, as those patients with MSI high (MSI-H) tumors had a more positive prognosis compared to MSI low (MSI-L) or Microsatellite stable (MSS) tumors [33]. Microsatellite status was obtained from the surgical pathology report based on immunohistochemistry (IHC) analysis evaluating expression of MSH2, MSH6, MLH1 and PMS2 proteins [34] in the 38 tumors examined for Rad51C variants. Among the 38 tumors, 34 were MSS tumors and four had MSI. Among the 34 MSS tumors, 16 tumors expressed variant 1, 11 variant 2, 13 variant 3, and seven had no expression of the variants. Of the four MSI tumors, two tumors expressed variants and the other two lacked expression of variants. Variant 1 was expressed in two tumors, while variant 2 and variant 3 were expressed in one tumor, which expressed all three variants. The two tumors with MSI without expression of Rad51C variants were low grade, moderately differentiated adenocarcinoma, and had absence of MLH1 and PMS2 protein expression.

### Rad51C Promoter methylation in colorectal tumors and cancer cells

**Figure 4:**
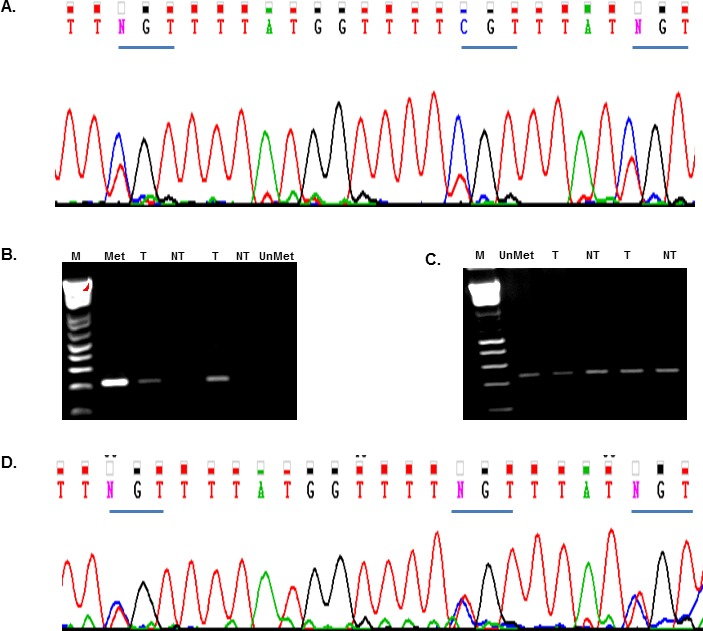
Rad51C Promoter methylation analysis The DNA was isolated from colorectal tumors and LS-174T cells pre and post 5-azacytidine treatment at a dose of 5μM for 72 hours. The DNA samples were bisulfite converted and PCR amplified using the primers that amplify the Rad51C promoter region (Supplementary Table S1). A total of 30 CpG sites were located in the product. The PCR amplification produced 408 bp product which is subjected to sequencing. A) The chromatogram for 408bp product showed methylation at three sites in Rad51C promoter region in colorectal tumors and LS-174T cells. B) The electrophoresis gel image of the methylation specific PCR (MS-PCR) amplification from tumors (T) and Non-tumors (NT) is shown. The Methylated DNA (Met) and Un-methylated DNA (UnMet) is used as controls. The MS-PCR showed methylation in tumors as compared to non-tumors. C) The electrophoresis gel image of the Un-methylated DNA. The PCR amplification of bisulfite converted DNA from tumors (T) and Non-tumors (NT) is shown. The Un-methylated DNA (UnMet) is used as control. D) The colorectal tumor cell LS-174T was treated with 5-azacytidine. Bisulfite sequencing showed de-methylation at the three sites of Rad51C promoter region.

We analyzed the promoter methylation pattern of the Rad51C tumor suppressor gene in the colorectal tumor cells. Bisulfite sequencing was used for the analysis. The promoter region of Rad51C is located on chromosome 17 (between nucleotide positions 56,769,721 and 56,770,129, sequence accession number NC_000017.11). DNA isolated from colorectal tumors and cells was bisulfite treated as described in Materials and Methods. The bisulfite treated DNA was PCR amplified to produce an amplicon fragment of 408 bp using the primers BiProF; BiProF, (Supplementary Table S1). The amplicon contains 30 CpG sites. The PCR amplified bisulfite treated DNA was sequenced (BiProSeqF, BiProSeqR; Supplementary Table S1). The sequence analysis of the bisulfite treated DNA from six colorectal tumors showed three sites that are methylated in the identical position in the promoter region of Rad51C (Fig. 4A). We then analyzed LS-174T colorectal tumor cells for Rad51C promoter methylation and found the same sites methylated as seen in the colorectal tumors. To evaluate the degree of methylation in the promoter region of Rad51C, we conducted methylation specific PCR (MS-PCR) analysis on 19 pair-matched tumor and non-tumor samples. Four of the 19 pairs contain detectable methylation in tumor samples only (Fig. 4B-C), 12 paired samples had no detectable methylation and three paired samples contain detectable methylation in both tumor and non-tumor by MS-PCR. The MS PCR primers for methylated and un-methylated PCRs are shown in the Supplementary Table S1.

### Effect of 5-azacytidine on the expression of Rad51C variants

In view of demonstration of several hypermethylated CpG sites within the promoter of Rad51C, we wonder if the expression of the observed Rad51C variants could be altered by treatment with DNA hypomethylating agents. To test this, we treated LS-174T cells with 5-azacytidine at 5μM for 72 hours. DNA was isolated and bisulfite treated. A 408bp PCR product was amplified using the primers BiProF and BiProR, (Supplementary Table S1). The PCR amplified products were gel extracted and subjected to sequencing using the primers, BiProSeqF and BiProSeqR (Supplementary Table S1). Sequencing analysis confirmed demethylation at three methylated CpG sites in the promoter region of Rad51C (Fig. 4D).

**Figure 5:**
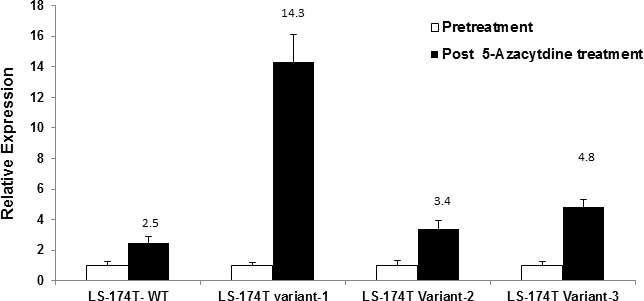
Real time PCR analysis of Rad51C variant expression in LS-174T colorectal tumor cells post 5-azacytidine treatment The LS-174T cells were treated with 5-azacytidine at dose of 5μM for 72 hours. The total RNA was isolated from pre and post 5-azacytidine treated LS-174T colorectal tumor cells and reverse transcribed to cDNA. The cDNA was then used as template for Rad51C variant expression analysis using real time specific primers and SYBR green dye (Supplementary Table S1). The analysis showed 14.3 fold increase in relative expression of RNA for variant 1, 3.4 folds for variant 2 and 4.8 folds for variant 3, and 2.5 folds increase for wild type.

To quantitatively analyze the effect of promoter methylation on RNA expression of Rad51C variants, RNA from azacytidine treated cells was reverse transcribed to cDNA and analyzed with SYBR green real time PCR. The real time PCR primers and respective amplicon sizes for variant 1, 2 and 3 are described in Supplementary Table S1. 18S ribosomal rRNA was used as endogenous control in separate reactions. The real time PCR analysis of LS-174T colorectal cells treated with 5-azacytidine, showed a 14.3 fold and 4.8 fold increase in the expression of variant 1 and 3, respectively in comparison to untreated controls. The short variant 2 showed a 3.4 fold increase in comparison to untreated controls. Wild type Rad51C had only a 2.5 fold increase post 5-azacytidine treatment compared to untreated controls (Fig. 5).

### Detection of Rad51C protein variants in colorectal tumor cells

We established the predicted protein sequence of the Rad51C variants by in silico translation using EMBOSS [35]. Variant 1 RNA sequence predicted 46 amino acids (aa) following the alternatively spliced exon-6 of Rad51C, resulting in a new chimeric protein with a predicted molecular weight of 41.6 KDa. The RNA sequence analysis for Rad51C variant 2, post exon -5, showed 24 additional aa and terminates the protein producing a 33.6 KDa protein. Rad51C variant 3 generates a premature stop codon terminating the protein at exon-9 with 4 aa post exon-6 (Supplementary Table S2).

**Figure 6:**
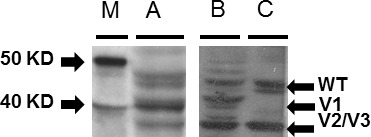
Western immunoblot analysis for Rad51C variant protein expression from colorectal tumors, matched non-tumors and LS-174T cells Protein was isolated from colorectal tumors, matched non-tumors and LS-174T cells. The protein was analyzed on 4-12% SDS page gel and blotted on nitrocellulose membrane. Rad51C wild type and variant proteins were detected using a mouse monoclonal Rad51C antibody. Western analysis showed the proteins from LS-174T cells contained a 47 KDa protein for wild type and a 41 KDa for predicted Rad51C variant 1 and 33 KDa for predicted variant 2 or 3 (A). The analysis of proteins from colorectal tumor showed similar variant expression pattern as in LS-174T tumor cells (B). The analysis of non-tumor protein showed expression of wild type and variant 2 or 3. The variant 1 was absent in non-tumor protein (C).

Protein was isolated from colorectal tumors, matched non-tumors and LS-174T cells (Fig. 6). The protein was size fractionated on 4-12% SDS page gel and blotted on nitrocellulose membrane. Rad51C wild type and variant proteins were detected using a mouse monoclonal Rad51C antibody that was predicted to recognize WT and all of the putative variants. Western immunoblot analysis showed that LS-174T cells contained a 47 KDa protein (wild type) and a 41 KDa protein as predicted for Rad51C variant 1, and a 33 KDa protein as predicted for variants 2 or 3. The analysis of proteins from colorectal tumors showed similar variant expression pattern as in LS-174T tumor cells. The analysis of non-tumor protein showed expression of wild type and variant 2 or 3. Variant 1 was not detected in non-tumor samples.

## DISCUSSION

Sequencing analyses have identified numerous recurrently mutated genes and chromosomal translocations in colorectal cancers [36, 37]. However, information regarding the expression of alternatively spliced gene variants in this malignancy is limited. Recent studies on the Rad51C gene have shown 14 germline sequence alterations among 1100 breast and ovarian tumor samples from patients [6]. Included in these germline alterations is an alternatively spliced isoform of Rad51C without exon-6. This isoform was shown to be involved in tumor cell proliferation [6]. Herein we report the identification of three Rad51C spliced transcript variants, in human colorectal tumors. Variant 1 was overexpressed in tumors.

All three RAD51C variants contained an intact N-terminus domain (exons 1-5). Several studies have shown DNA dependent ATPase activity in the region between exon 2 to exon 5 at aa 125 to132 of Rad51C [10, 17]. Rad51C N-terminus domain is known to form complexes with Rad51B, D and XRCC2 [13, 14], thus variant 1 maintains protein to protein interactive functions. We would hypothesize then that overexpression of variant 1 should stabilize the complex and facilitate DNA repair and replication. To evaluate the biological function of the variant 1, we performed *In vitro* transient overexpression of Rad51C variant 1 in HCT116 colorectal tumor cells (Supplementary Figures S2). A 1.8 fold increase in the cell proliferation was recorded in the variant 1 expressing cells as comparing to empty vector transfected controls according to BrdU FACS analysis (Supplementary Figures S3). In addition we evaluated the expression of Rad51C variant 1 in cell proliferation with immunofluorescence staining after labeling with BrdU. We found the cells expressing Rad51C variant 1 had more BrdU positive cells as comparing to cells transfected with empty vector and non-transfected cells (Supplementary Figures S4). These *In vitro* data suggest the Rad51C variant 1 may promote cell proliferation in colorectal tumors.

Promoter methylation of Rad51C has been shown in breast and ovarian cancer patients [38]. Here we show promoter methylation of Rad51C in colorectal tumor and LS-174T DNA samples. DNA methylation is known to exert multiple functions including regulation of gene expression, determining alternate poly A choice and tissue specific selection of alternate promoters [39]. It has been shown that Methyl CpG (MECP2) binding proteins are enriched in alternatively spliced exons and chemical or genetic disruption ablates the aberrant exon skipping events [40]. Our results show that the Rad51C variants expression was up regulated after treatment with 5-azacytidine, a classic demethylation agent. This indicates that Rad51C promoter methylation can regulate the expression of variants in colorectal tumors. Further investigation is needed to elucidate differential function of the Rad51C variant 1, given its association with the malignant phenotype, and its higher degree of response to demethylation agents. Experiments to evaluate its potential effect in drug resistance and DNA repair are under way in our laboratory.

## MATERIALS AND METHODS

### Patient tumor tissue samples collection

The human tumor tissue samples were obtained from the tissue procurement shared resources of The Ohio State University Comprehensive Cancer Center, after institutional review board approval. Tumor characteristics such as histology, tumor site and size as well as microsatellite stability parameters were obtained from the pathology report. The processed samples were excised into formalin fixed and paraffin embedded (FFPE) and frozen samples. The frozen samples were stored in a −80^°^C freezer for RNA and DNA extraction.

### RNA and DNA analysis

The total RNA and DNA were isolated by homogenization and sequential precipitation following the protocol from TRIzol reagent (Life Technologies, CA). The RNA and DNA obtained from the colorectal tumor tissue samples was quantified at absorbance 260nm and 280 nm using Nanodrop 2000C (Thermoscientific, DE).

### RT-PCR

The RT-PCR primers were designed using the automated Primer3 [41]. The RT-PCR was performed in 25 μl reaction containing final concentration (1x) of 5μl of 5x Qiagen One-step RT-PCR buffer, 400 μM of each dNTPs, 0.6 μM each of forward and reverse primers, 1μl of Qiagen One step RT-PCR enzyme mix and template RNA up to 400ng and volume made up to 25 μl using PCR grade water. The cDNA of about 200ng was PCR amplified for 30 min at 54^°^ C, initial heat activation for 15 min at 95^°^ C, 33 cycles of initial denaturation for 30 sec at 94^°^ C, annealing for 30 sec at 61^°^ C, extension for 30 seconds at 72^°^ C, final extension for 5 min at 72^°^ C. The forward and reverse primers used for RT-PCR and their amplicon sizes are mentioned in (Supplementary Table S1). To identify each of the variant, variant specific primers that span exon junctions were designed. All primers are listed in the Supplementary Table S1.

### FANCD2 foci identification

The FA triple staining immunofluorescence method previously developed in our laboratory [32] was used to assess the FANCD2 foci in colorectal tumor tissues.

### 5-azacytidine (5-aza) treatment of cells

Human colon cancer cell lines LS-174T purchased from ATCC^®^ were grown in complete EMEM medium with 10% fetal bovine serum and 1% penicillin/streptomycin at 37^°^C (5%, CO_2_). For the treatment of cells, 5-azacytidine was filter-sterilized and added directly to the fresh cell culture media. Fresh 5-azacytidine was added every 24 hours at dose of 5μM for 72 hours. The post treatment cells were lysed, and the pellets were collected and stored at −80^°^C.

### Bisulfite sequencing and promoter methylation

Bisulfite conversion of genomic DNA from colorectal tumors and cells was performed with EpiTect 96 Bisulfite kit (Qiagen, Hilden, Germany). The promoter region of Rad51C was retrieved from the Ensemble browser (http://www.ensembl.org/Homo_sapiens/). The prediction of CpG islands and PCR primer sequences were designed using Meth primer program [42]. PCR amplification of the bisulfite DNA was performed in 25 μl reaction using 1x methylation specific PCR buffer, 10mM of dNTP mix, 0.4 μM of each forward and reverse primers, 1 μl of (~150ng) of bisulfite treated DNA sample and 0.4 μl of 1.0 unit Platinum ® Taq DNA polymerase following the protocol from (Invitrogen). PCR was carried out with initial heat denaturation step of 95^°^C for 10 min, 38 cycles of 95^°^C for 30 s, annealing at 61^°^C for 30 s, extension at 72^°^C for 30 s and final extension step at 72^°^C for 5 min. The amplified products were gel extracted and purified using the QIAquick Gel Extraction Kit (Qiagen, CA) and subjected to sequencing using primers mentioned in (Supplementary Table S1). The Methylation specific PCR (MS-PCR) was carried out with EpiTect MSP kit (Qiagen, CA) using the 2X master mix following the manufacturer recommendations. The PCR was carried out with initial heat denaturation step of 95^°^C for 10 min, 38 cycles of 95^°^C for 15 s, annealing at 51^°^C for 30 s, extension at 72^°^C for 30 s and final extension step at 72^°^C for 5 min.

### Real time PCR

The RNA expression levels of the Rad51C variants were analyzed by SYBR green real time PCR. The RNA isolated from colorectal tumor cells were treated with 5-azacytidine and reverse transcribed to cDNA using Superscript^TM^ II Reverse Transcriptase from Invitrogen (CA, USA). The primers for real time PCR amplification were designed using Primer3 software [41]. The SYBR green real time Quantitect PCR kit was purchased from Qiagen Sciences (Maryland, USA). The primers used for real time PCR amplification are mentioned in Supplementary Table S1. The cDNA was used as template and the final reaction consisted of 1x SYBR green master mix, 10pmol/μl of forward and reverse primers. Also human 18S rRNA is used as internal control in separate reactions.

### Immunoblot

Equal amount of proteins were size-fractionated on 4-12% NuPAGE. Proteins were then transferred onto nitrocellulose membrane. The membrane was blocked with blocking buffer (5% nonfat milk). The blocked membrane was then incubated with target mouse monoclonal Rad51C antibody (Sigma Aldrich^®^, MO) at 4^°^C overnight. After washing the membrane with TBS-T (20mM Tris, 0.9%Nacl) for three times 30 minutes each, the membrane was then incubated with anti-mouse secondary antibody (Sigma Aldrich^®^, MO) for 1 h at room temperature. After washing five times, 10 minutes each, a chemiluminescent detection system (ECL western blotting detection reagents, GE Healthcare) was used to detect the secondary antibody. Finally the membranes were exposed to x-ray films.

## SUPPLEMENTARY MATERIALS, FIGURES AND TABLES


