# Supplementary material for: Overexpression of Rad51C splice variants in colorectal tumors
Source: Oncotarget. 2014 Dec 30;6(11):8777–87. doi: 10.18632/oncotarget.3209 (PMC4496183; doi:10.18632/oncotarget.3209)
Supplement: Supplementary file 1 [file oncotarget-06-8777-s001.pdf]

Overexpression of Rad51C splice variants in colorectal tumors

Supplementary Methods and Materials

Transfection

HCT116 cells were grown at a density of 10^6/ml in McCoy 10% FBS complete medium. The vector PIRES2-eGFP (PT3267-5) was linearized with ECORI enzyme and ligated with Rad51C variant 1 following standard ligation protocol from New England Biolabs. The plasmid Rad51C variant 1 co-expressing eGFP or control eGFP empty vector alone were transiently transfected using Lipofectamine 2000 (Life technologies, CA) in a 6 well plate. Expression of eGFP was confirmed with a fluorescence microscope.

Flow cytomtery

HCT116 Cells transfected with Rad51C variant 1 eGFP or eGFP vector alone were incubated in 10^6/ml in Mccoy/10% FBS. After 48 hours, BrdU (5-Bromo-2′-deoxyuridine) (Sigma Aldrich, MO) was added to the culture medium at final concentration of 10µm for 4 hrs. The cells were sorted for eGFP using BD FACSAria (BD Biosciences). The cells were collected and probed using primary mouse anti-BrdU antibody (Cell signaling, MA), fixed and permeabilized using cytofix/cytoperm buffer, treated with 30 µg of DNase I and then stained with Anti mouse Alexa flour 555 anti-BrdU antibody (Life technologies, CA). Analysis was carried out on a BD FACSCalibur™ platform (BD Biosciences) equipped with two lasers an air-cooled argon laser and a red diode laser.

Immunofluorescence

HCT116 cells were grown in a 6 well plate containing cover slips made of Poly-L-Lysine BD BioCoat (BD Biosciences, MA) at a density of 10^6/ml in McCoy 10% FBS complete medium. The Rad51C variant 1 which co-express eGFP or control eGFP vector alone were transiently transfected using Lipofectamine 2000 (Life

technologies, CA). After 48 hours, BrdU was added to the culture medium at final concentration of 10μm for 4 hours. The cells were fixed with 4% Paraformaldehyde and permeabilized with Triton –x-100 at 0.3%. The cells were then incubated with Primary mouse BrdU antibody (Cell signaling, MA) overnight and rinsed with PBS and labeled with Alexa Flour 594 donkey anti-mouse IgG secondary antibody (Life technologies, CA) and mounted with Vectasheild medium with DAPI H-1500 (Vector Laboratories Inc. CA).

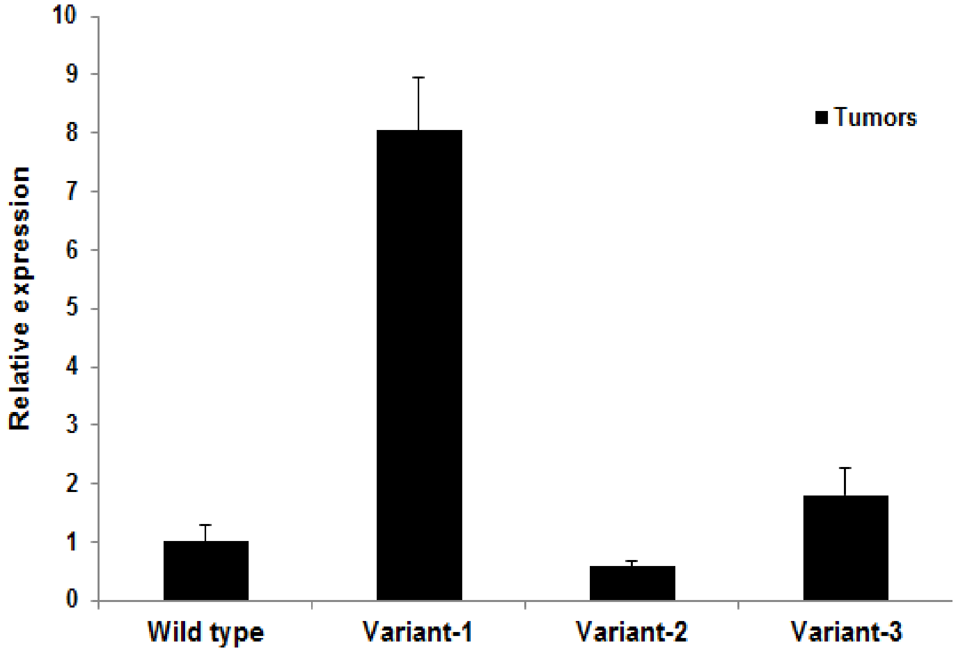

Supplementary Figure S1: Real time PCR analysis of Rad51C variant expression in colorectal tumors. The total RNA was isolated from 9 colorectal tumors and reverse transcribed to cDNA. The cDNA was then used as template for Rad51C variant expression analysis using the real time specific primers and SYBR green dye (Supplementary Table S1). On average variant 1 was expressed 8.05 fold higher as compared to wild type. Variants 2 and 3 were expressed at lower levels.

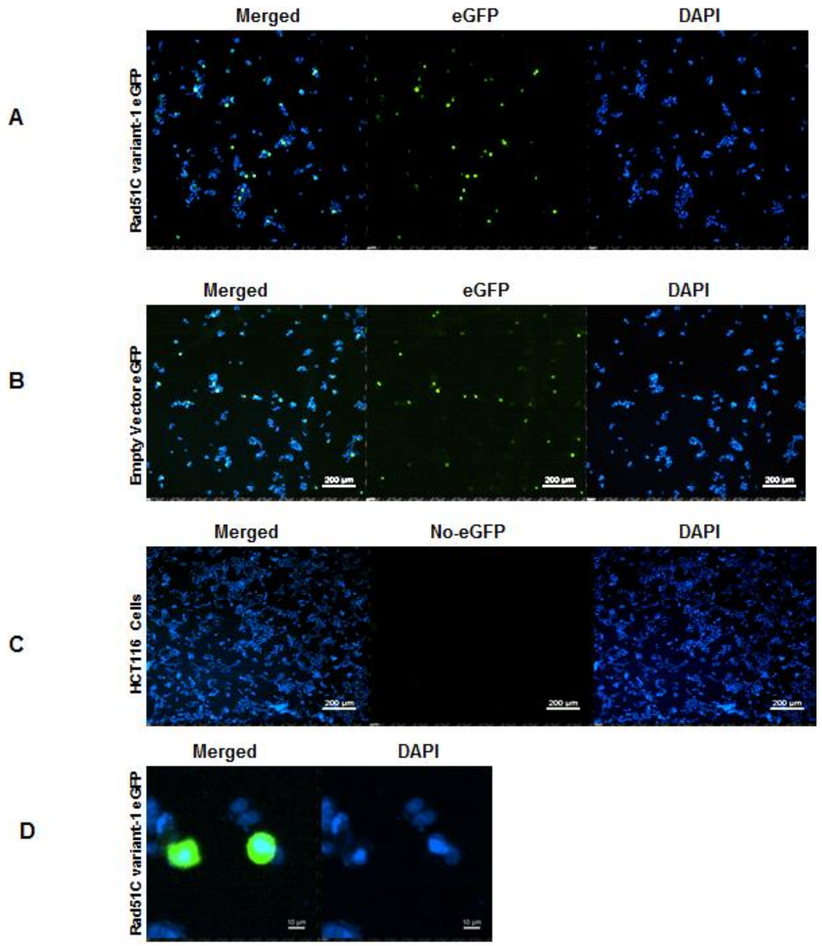

Supplementary Figure S2: Rad51C-variant-1 co-expression with eGFP and co-staining by 4',6-Diamidino-2-Phenylindole (DAPI). HCT116 cells were transiently transfected with plasmid Rad51C variant-1 co-expressing eGFP protein. After 48 h, cells were fixed and stained with 4% Paraformaldehyde and mounted with Vectasheild medium with DAPI H-1500. A) The fluorescent image of nuclei co-stained with DAPI (blue) for cells transfected with Rad51C variant-1 co-expressing eGFP (green) is shown. B) Nuclei stained with DAPI in cells co-expressing eGFP protein only used as control; C) HCT116 cells with no transfection is used as negative control; D) A magnified image of nuclei co-stained with DAPI (blue) for cells transfected with Rad51C variant-1 co-expressing eGFP (green) is shown.

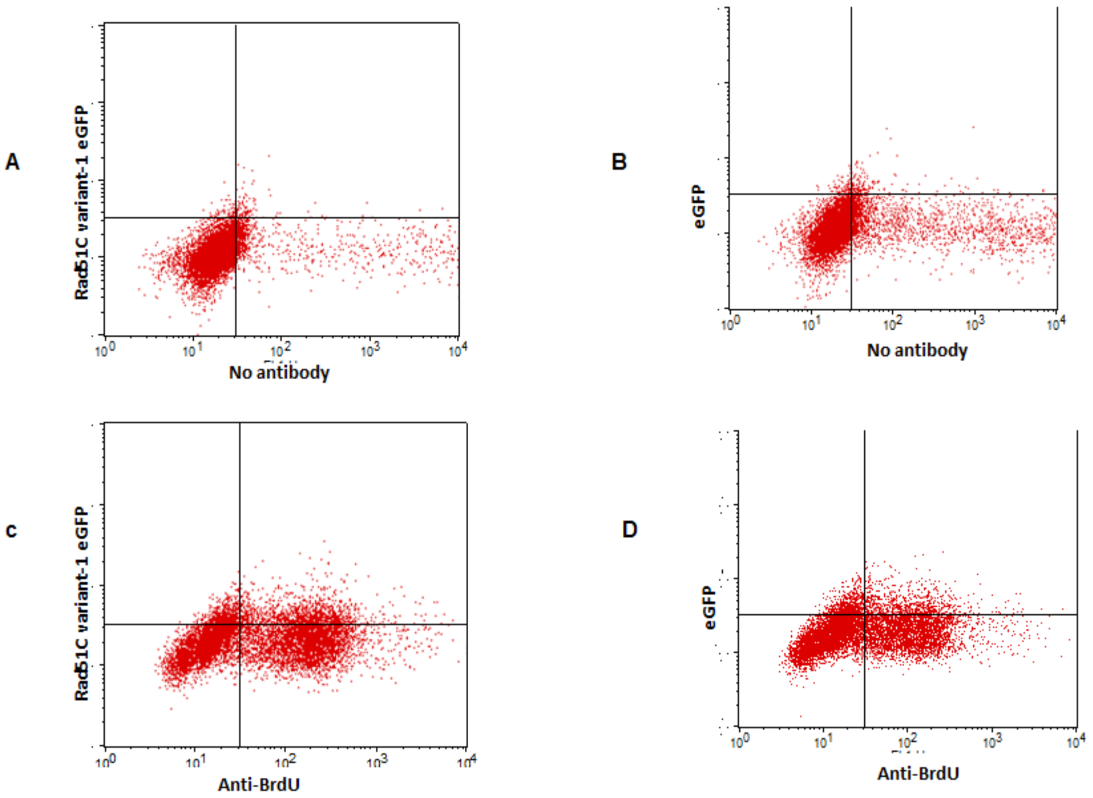

Supplementary Figure S3: Cell proliferation by Flow cytomtery analysis. HCT116 Cells transfected with Rad51C- variant-1 eGFP or eGFP empty vector alone at 5µg of DNA and were incubated in 10^6/ml in Mccoy/10% FBS. After 48 hours, BrdU (5-Bromo-2′-deoxyuridine) at final concentration of 10µm was added to the culture medium and incubated for 4 hrs. The cells were sorted for eGFP using FACS. The cells were then collected and probed with mouse primary anti-BrdU antibody in 50µl staining buffer (1xDPBS+3%Fetal Bovine Serum) following the protocol from BD bioscience. The cells were fixed and permeabilized using cytofix/cytoperm buffer and stained using secondary anti-mouse Alexa Flour 555 anti-BrdU antibody. A) The HCT116 cells transfected with Rad51C variant-1 treated with BrdU was used as negative control (without anti-BrdU antibody); B) The HCT116 cells transfected with empty vector treated with BrdU was used as negative control (without anti-BrdU antibody); C) The Cells transfected with Rad51C variant-1 were gated on the basis of forward- and side-scatter and the cell proliferation rate was determined to be 1.8 folds higher in Rad51C variant-1 expressing cells as compared to D) eGFP expressing cells.

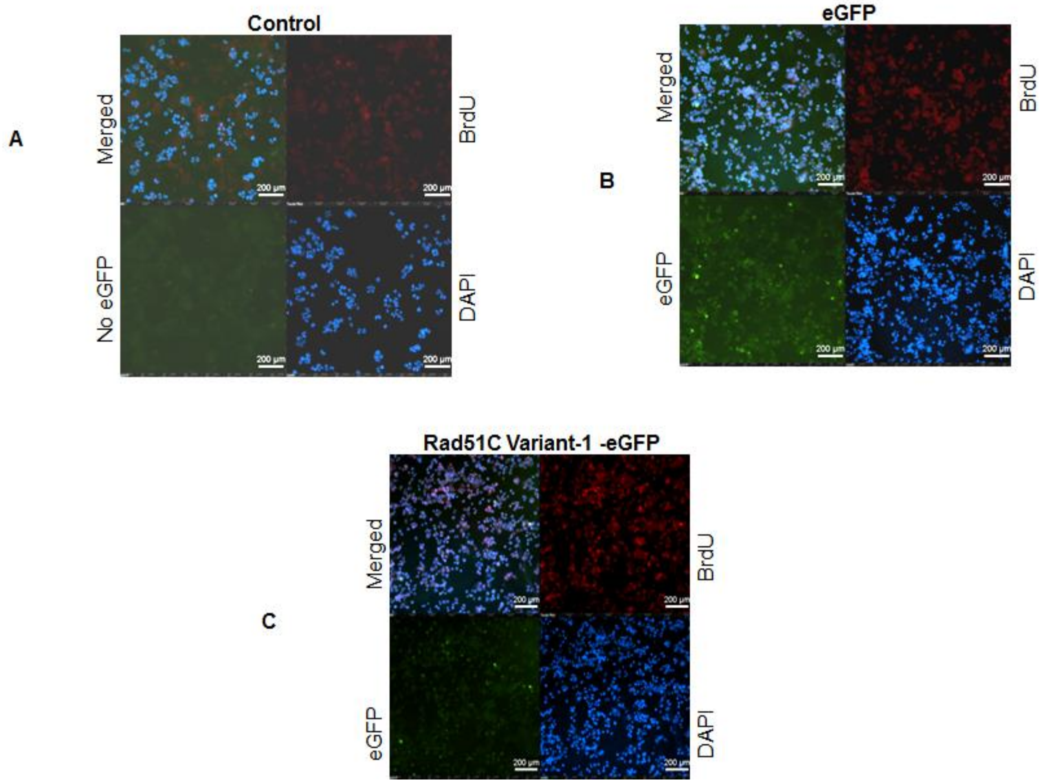

Supplementary Figure S4: HCT116 cells were transfected with Rad51C- variant-1 or empty vector. After 48 hours, BrdU at final concentration of 10µm was added to the culture medium and incubated for 4 hours. The cells were fixed and labeled with primary mouse BrdU antibody and Alexa Flour 594 donkey anti-mouse IgG secondary antibody and mounted with Vectasheild medium with DAPI H-1500 to visualize the nuclei. A) The HCT116 cells used as negative control. B) Cells transfected with Empty vector; C) Cells transfected with Rad51c variant-1. The cell expressing eGFP is shown in green, co-staining with DAPI is indicated in blue and BrdU is indicated as Red.

Supplementary Table S1: Rad51C primers

S.No	Rad51C	Primer Name	Primers	Sequence 5' to 3'	Amplicon size (bp)
a	**RNA**	ERF<br>ERR	PCR and sequencing Forward<br>PCR and sequencing Reverse	5'-TGGTATTGCTTTTCCATTTCG-3'<br>5'-TTTGCAATGAACATGCAGAAG-3'	380
b	**Variant-1**	E-7F<br>E-7R	RT-PCR Forward probe<br>RT-PCR Reverse probe	5'- TGCTTGTTCCTGCATTAGGTT-3'<br>5'-CCACTTGTACACATTGATTTCACA-3'	236
c	**Variant-2**	E-6-7F<br>E-6-7R	RT-PCR Forward probe<br>RT-PCR Reverse probe	5'-GCAAATAATCACAGATTAGCTGTT -3'<br>5'-TCATTCATGCCATAGTGTGTTT-3'	280
d	**Varaint-3**	E-7-8F<br>E-7-8R	RT-PCR Forward probe<br>RT-PCR Reverse probe	5'-GCTTGTTCCTGCATTAGCCT -3'<br>5'-TCATTCATGCCATAGTGTGTTTA-3'	219
e	**Bisulfate DNA Promoter region Chromosome 17 56,769,721-56,770,129**	BiProF<br>BiProR	Forward<br>Reverse<br>Sequencing Forward<br>Sequencing Reverse	5'-GGAGAATTTATTGGGTTTGGTTT-3'<br>5-TCACCTCTAAAAATTCCTCAACAAT-3'<br>5'-GAATTTATTGGGTTTGGTTTTT-3'<br>5'-TCACCTCTAAAAATTCCTCAACAAT-3	408 (with 30 CpG)
f	**Real time Variant-1**	RE-7F<br>RE-7R	Forward<br>Reverse	5'-TGGTATTGCTTTTCCATTTCG-3'<br>5'-TTGTTTCTGGGTTATAATTCTTCC-3'	187
g	**Real time Variant-2**	RE-6-7F<br>RE-6-7R	Forward<br>Reverse	5'-TGCTTGTTCCTGCATTAGGTT-3'<br>5'-TTGTTTCTGGGTTATAATTCTTCC-3'	190
h	**Real time Varaint-3**	RE-7-8F<br>RE-7-8R	Forward<br>Reverse	5'-GCTTGTTCCTGCATTAGCCT-3'<br>5'-TCATTCATGCCATAGTGTGTTTA-3'	130
i	**Methylation Specific PCR**	MSP-F<br>MSP-R	Forward<br>Reverse	5'-TTcGTTTTATGGTTTTcGTTTATcG-3'<br>5'-CCCTCGCTAAAACGTACGAC-3'	235
j	**Un-methylation Specific PCR**	UMSP-F<br>UMSP-R	Forward<br>Reverse	5'-TTtGTTTTATGGTTTTtGTTTATtG-3'<br>5'-CCCTCGCTAAAACGTACGAC-3'	235

Supplementary Table S2: Translation of Rad51C Variants

Rad51C Variant	Sequence with Amino acid	mRNA Length	Total Amino acid	Predicted Molecular weight of protein
**Rad51C variant-1**	Rad51C (1 to 6)- <br>mrgktfrfem qrdlvsfpls pavrvklvsa gfqtaeelle vkpselskev giskaealet lqiirreclt nkpryagtse shkkctalel leqehtqgfi itfcsalddi lgggvplmktteicgapgvgktqlcmqlavdvqipecfggvageavfidt egsfmvdrvv dlataciqhl qliaekhkge ehrkaledft ldnilshiyy frcrdytell aqvyllpdfl sehskvrlvivdgiafpfrh dlddlslrtr llnglaqqmi slannhrlav iltnqmttki drnqallv palgwqhctshparrnaqycfksnlrdlEILLLLLHVHCKQKVPEHPETVTRPR GRIITQKQISKCTNLLML Stop	1157 bp	385	41.6 KDa
**Rad51C variant-2**	Rad51C (1 to 5) – <br>mrgktfrfem qrdlvsfpls pavrvklvsa gfqtaeelle vkpselskev giskaealet lqiirreclt nkpryagtse shkkctalel leqehtqgfi itfcsalddi lgggvplmkt teicgapgvg ktqlcmqlav dvqipecfgg vageavfidt egsfmvdrvv dlataciqhl qliaekhkge ehrkaledft ldnilshiyy frcrdytell aqvyllpdfl sehskvrlvi vdgiafpfrh dlddlslrtr llnglaqqmi slannhrlav G N I V Q A T Q P E G M H S T V S N Q T S G I Stop	951 bp	317	33.6 KDa
**Rad51C variant-3**	Rad51C (1 to 6) – <br>mrgktfrfem qrdlvsfpls pavrvklvsa gfqtaeelle vkpselskev giskaealet lqiirreclt nkpryagtse shkkctalel leqehtqgfi itfcsalddi lgggvplmktteicgapgvgktqlcmqlavdvqipecfggvageavfidt egsfmvdrvv dlataciqhl qliaekhkge ehrkaledft ldnilshiyy frcrdytell aqvyllpdfl sehskvrlvivdgiafpfrh dlddlslrtr llnglaqqmi slannhrlav iltnqmttki drnqallv palSGI Stop	954 bp	318	33.8 KDa
